# TAFRO Syndrome: A Case of Significant Endocrinopathy in a Caucasian Patient

**DOI:** 10.7759/cureus.4946

**Published:** 2019-06-19

**Authors:** Jennifer L Miatech, Nipur R Patel, Nicholas Q Latuso, Pavani K Ellipeddi

**Affiliations:** 1 Baton Rouge General Internal Medicine Residency Program, Baton Rouge General Medical Center, Baton Rouge, USA; 2 Hematology & Oncology, Baton Rouge General Medical Center, Baton Rouge, USA

**Keywords:** tafro, castleman, endocrinopathy, poems, lymphoproliferative, cytokine, siltuximab, vegf, imcd, caucasian

## Abstract

Idiopathic multicentric Castleman disease (iMCD) is a lymphoproliferative disorder that manifests as multiorgan dysfunction secondary to widespread inflammation. The underlying pathogenesis is driven by an excessive and inappropriate cytokine storm. TAFRO syndrome is a rare subtype of iMCD, characterized by thrombocytopenia, anasarca, myelofibrosis, renal dysfunction, and organomegaly. Multiorgan dysfunction is a known consequence of this syndrome, although endocrine involvement has yet to be reported. We present a case of TAFRO in a previously healthy Caucasian male who presented with abdominal pain, dysuria, diffuse anasarca, and ascites. On presentation, the patient was found to have acute kidney injury, thrombocytopenia, elevated inflammatory markers, elevated interleukin-6 (IL-6), and endocrinopathy. Following an extensive infectious and autoimmune workup, lymph node biopsy confirmed the diagnosis of TAFRO. The patient was started on prednisone, rituximab, and anti-IL-6 therapy with siltuximab. He achieved clinical remission after 4 months of treatment, with normalization of renal function, thrombocytopenia, inflammatory markers, and endocrinopathy. He has continued on siltuximab for maintenance therapy. It is our hope that this unique case of TAFRO syndrome with significant endocrinopathy will add to the growing literature surrounding iMCD, and help clinicians better understand the pathogenesis and treatment of this rare disease.

## Introduction

Multicentric Castleman disease (MCD) is a rare lymphoproliferative disorder that results in systemic inflammation and multiorgan dysfunction. MCD is further classified as idiopathic multicentric Castleman disease (iMCD) when there is no association with human herpesvirus 8 (HHV-8). The estimated incidence of iMCD in the United States is between 1000 and 1500 cases [1]. TAFRO syndrome, a distinct subtype of iMCD, has predominantly been described in Japanese populations. TAFRO syndrome is characterized by thrombocytopenia, anasarca, myelofibrosis, renal dysfunction, and organomegaly.

TAFRO presents a diagnostic challenge to clinicians given the overlap with several other disease states that can mimic the clinical presentation. Diseases that can manifest with a similar constellation of symptoms include autoimmune disorders, infections, malignancies, and POEMS syndrome. POEMS is a monoclonal plasma cell disorder defined by polyneuropathy, organomegaly, endocrinopathy, monoclonal protein elevation, and skin changes [2]. Although endocrinopathy is a key feature of POEMS, it is not an established clinical finding in patients with TAFRO. While these two syndromes are distinct entities, they both demonstrate hypercytokinemia facilitated by several of the same cytokines, resulting in a potential for diagnostic complexity.

Although several cytokines have been implicated in the pathogenesis, the complete mechanism remains to be elucidated. Given the rarity of the disease and the fairly recent clinical classification, there is a paucity of reported cases in the literature. We present a case of TAFRO syndrome in a Caucasian male with the rare clinical manifestation of multiple endocrinopathies.

## Case presentation

A previously healthy 21-year old Caucasian male presented to our hospital with complaints of abdominal pain, dysuria, and increased urinary frequency. One week prior to admission, he was diagnosed with a urinary tract infection at an outpatient facility and was treated with antibiotics. He had no other medical history and was in otherwise good health. The patient had no significant family history. At presentation, he had a temperature of 98.9°F, heart rate of 111 beats per min, respiratory rate of 20 breaths/minute, blood pressure of 108/74 mmHg, and oxygen saturation of 100% on room air. Physical exam revealed tense ascites, diffuse anasarca, and bilateral axillary and right inguinal lymphadenopathy measuring less than 2 cm.

Initial laboratory testing revealed a normal white blood cell count of 9.52 K/uL with 73% neutrophils and 12% lymphocytes, microcytic anemia (hemoglobin 11.9 g/dL, MCV 77 fl), thrombocytopenia (platelet count 20 K/uL), elevated lactate dehydrogenase (275 U/L), and C- reactive protein (18.80 mg/dL). Peripheral smear showed microcytosis with no fragmented red blood cells or immature blast cells. The ADAMTS-13 activity was normal, and hemolytic workup was negative including direct Coombs testing, D-dimer, and haptoglobin; with mild elevations in the prothrombin time (PT), partial thromboplastin time (PTT), and international normalized ratio (INR) (Table 1). Iron studies revealed an iron of 15 ug/dL, the total iron binding capacity of 135 ug/dL, ferritin of 697 ng/mL, and percent saturation of 12%). Vitamin B12 (300 pg/mL) and folate (4.6 nm/mL) levels were near the lower limit of normal, with an elevated methylmalonic acid (950 nmol/L). The patient received iron, vitamin B12, and folate supplementation.

**Table 1:** Admission Laboratory Findings WBC = white blood cell; BUN = blood urea nitrogen; ALT = alanine aminotransferase; AST = aspartate aminotransferase; ALP = alkaline phosphatase; LDH = lactate dehydrogenase; CRP = C-reactive protein; PT = prothrombin time; PTT = partial thromboplastin; INR = international normalized ratio.

Laboratory studies	Admission	Reference range
Complete Blood Count		
WBC	9.52	4.0-11.0 K/uL
Hemoglobin	11.9	13.7-17.0 g/dL
Hematocrit	37.6	40.0-50.0%
Platelet count	20	150-400 K/uL
Blood Chemistry		
BUN	91	7-18 mg/dL
Creatinine	3.42	0.70-1.20 mg/dL
Calcium	8.3	8.5-10.1 mg/dL
Sodium	132	136-145 mmol/L
Potassium	4.9	3.5-5.1 mmol/L
Albumin	1.6	3.4-5.0 g/dL
Protein, Total	6.4	6.4-8.6 g/dL
ALT	7	16-61 U/L
AST	22	15-37 U/L
ALP	152	45-117 U/L
Bilirubin, Total	1.1	0.2-1.0 mg/dL
LDH	275	84-246 U/L
CRP	18.8	0.00-1.00 mg/dL
Uric acid	13.1	3.5-7.2 mg/dL
Coagulation		
PT	15.9	12.3-14.6 sec
PTT	37	23-36 sec
INR	1.3	0.8-1.2
Haptoglobin	310	300-200 mg/dL
Fibrinogen	857	225-450 mg/dL
D-Dimer	10.01	0.0-0.49 mg/L

**Figure 1:**
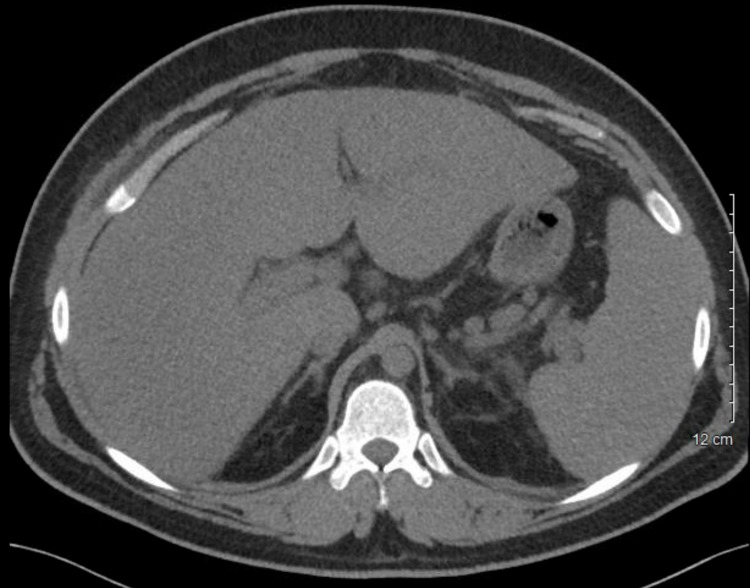
Non-contrast abdominal computed tomography obtained on admission showing hepatomegaly and splenomegaly.

The patient was also found to have an elevated blood urea nitrogen (91 mg/dL), creatinine (3.42 mg/dL), and hypoalbuminemia (1.6 g/dL). Uric acid levels were elevated, and he was started on rasburicase (Table 1). Urinalysis was significant for microhematuria with 6-10 red blood cells/hpf. Blood cultures were negative. Further workup on presentation included CT abdomen demonstrating hepatomegaly (25 cm), splenomegaly (18 cm), diffuse adenopathy, marked ascites, and small bilateral pleural effusions (Figure 1). Due to symptomatic ascites, a large volume paracentesis of approximately 8 L was performed, which revealed a serum albumin ascites gradient (SAAG) of <1.1, with normal cytology and cell count.

Additionally, the patient was found to have low testosterone of 26 ng/dL and follicle stimulating hormone (FSH) of <0.2 mIU/mL with hyperprolactinemia (prolactin 32 ng/mL), suggestive of central hypogonadism. Furthermore, subclinical hypothyroidism (thyroid stimulating hormone 1.040 uIU/mL, free triiodothyronine 0.91 pg/mL) as well as low vitamin D, 25-hydroxy levels (9.9 ng/mL) with elevated parathyroid hormone (163 pg/mL) was present (Table 2). MRI head was negative for obvious pituitary involvement.

**Table 2:** Endocrine Laboratory Results TSH = thyroid stimulating hormone; PTH = parathyroid hormone; FSH = follicle stimulating hormone.

Endocrine Studies	Admission	6 months	Reference range
Triiodothyronine, Free (T3)	0.91	2.45	2.18-3.98 pg/mL
Thyroxine, Free (T4)	0.65	1.04	0.76-1.46 ng/dL
TSH	1.04	2.29	0.358-3.740 uIU/mL
PTH	163.2	13.5	14.0-72.0 pg/mL
FSH	<0.2	6.85	1.4-18.1 mIU/mL
Prolactin	32	19	2.1-17.7 ng/mL
Testosterone, total	26	459	264-916 ng/dL

Autoimmune workup including antinuclear antibody (ANA) and antineutrophil cytoplasmic antibodies (ANCAs) was negative. Serum immunoglobulins and complement were within normal ranges, except for mildly low immunoglobulin G (IgG) levels. Infectious workup was negative, including human immunodeficiency virus (HIV), hepatitis A, hepatitis B, hepatitis C, Epstein-Barr virus (EBV), cytomegalovirus (CMV), and human herpesvirus 8 (HHV-8). Flow cytometry revealed normal findings.

Given the complexity of this case and presentation, many differential diagnoses were entertained, including POEMS syndrome. However, further workup revealed a normal serum protein electrophoresis (SPEP) without an M-spike, normal VEGF levels, and absence of osteosclerotic bone lesions on postiron emission tomography/computed tomography imaging.

TAFRO syndrome was suspected with the clinical presentation of anasarca, ascites, hepatosplenomegaly, and lymphadenopathy; along with the laboratory findings of thrombocytopenia, anemia, elevated inflammatory markers, and renal dysfunction. Treatment was initiated with oral prednisone 100 mg and rituximab 375 mg/m^2^ early during hospitalization while awaiting lymph node biopsy results. During this time the patient experienced progressive renal failure and anasarca, requiring hemodialysis for one week during hospitalization for significant volume overload. Multiple platelet transfusions and 1 dose of intravenous immunoglobulin (IVIG) 1 g/kg were given due to persistent thrombocytopenia with a nadir of 8 K/uL. A gradual rise in the platelet counts was seen after one week of prednisone and rituximab treatment.

**Figure 2:**
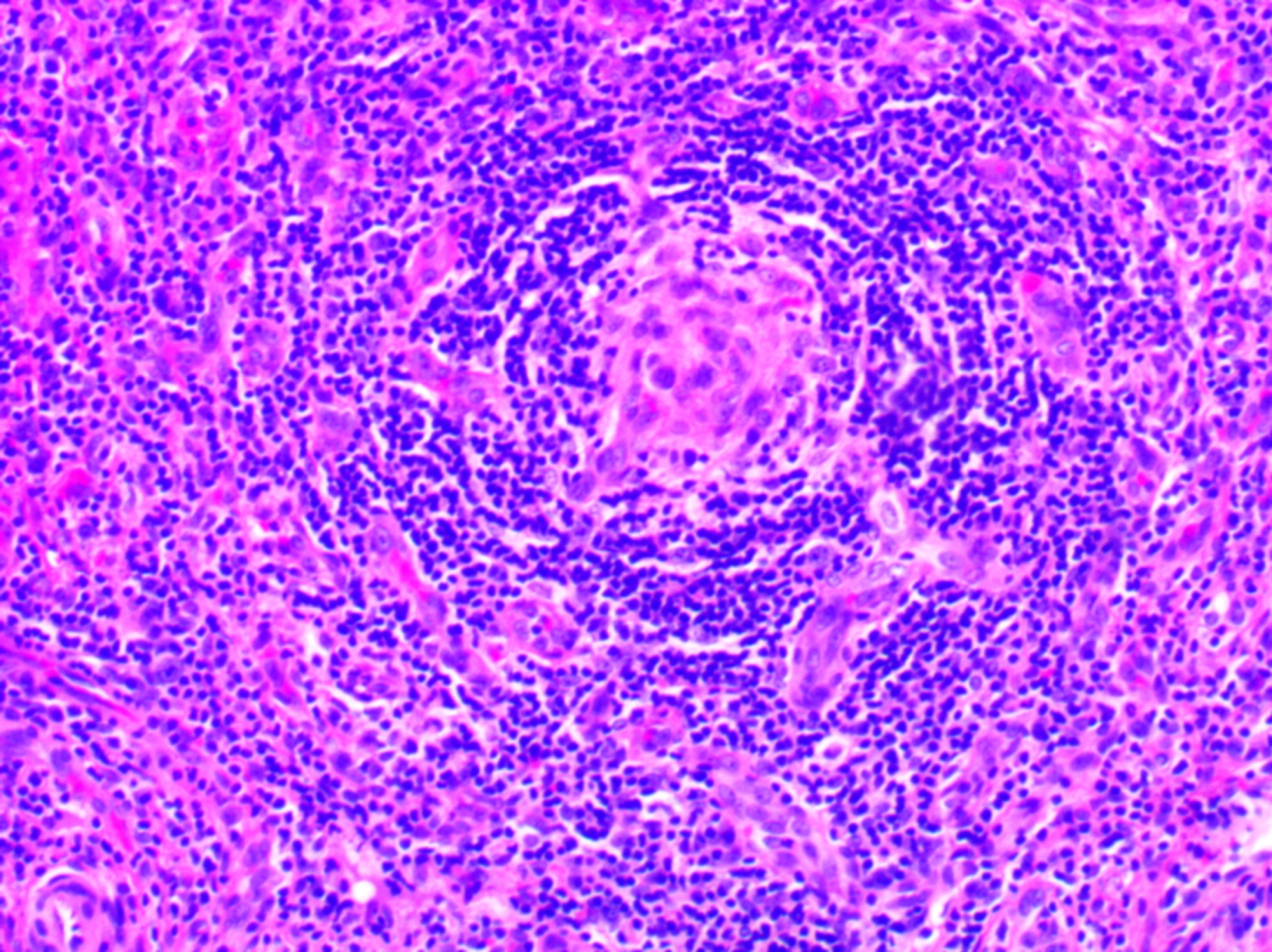
Histopathology of the right-sided inguinal lymph node showing marked interfollicular vascular proliferation with some regression and atrophy of the lymphoid follicles. In some areas, “onion-skinning” of the mantle cells is seen around the follicles with penetration by small blood vessels. Hematoxylin and eosin staining.

Bone marrow biopsy revealed a hypercellular marrow (95% cellularity) with myeloid hyperplasia, megakaryocytic hyperplasia, and dysmegakaryopoiesis. Biopsy of the right inguinal lymph node revealed marked interfollicular vascular proliferation, regression and atrophy of the lymphoid follicles, and onion-skinning of the mantle cells. These histopathological findings were consistent with the hypervascular subtype of Castleman disease (Figure 2). LANA-1 staining for HHV-8 was negative. Further testing revealed an elevated interleukin-6 (53.9, reference 0.0-15.5 pg/mL) with normal VEGF (111, 1-1115 pg/mL) and interleukin-2 (<31.2, reference 0.0-31.2 pg/mL) levels.

 

The final diagnosis of TAFRO syndrome was confirmed after lymph node biopsy revealed histopathological findings consistent with iMCD. The patient was then started on anti-IL-6 therapy with siltuximab at 11 mg/kg. The patient was discharged home on oral prednisone 20 mg once daily, rituximab 375 mg/m^2^ once weekly for a total of 8 doses, and siltuximab 11mg/kg once every 3 weeks. He continued to have significant ascites requiring daily furosemide and placement of an intraperitoneal catheter for biweekly drainage. With treatment, he experienced a gradual improvement in platelet counts, renal function, inflammatory markers, and resolution of his ascites. Thyroid and vitamin D levels were corrected with levothyroxine 75 mcg daily and cholecalciferol, respectively, prior to discharge. However, the central hypogonadism and hyperprolactinemia persisted upon discharge. After 4 months of anti-IL6 therapy, FSH, prolactin, and testosterone levels gradually normalized (Table 2). Clinical remission has been maintained for 6 months, and he continues siltuximab monotherapy to prevent remission.

## Discussion

In 2010 a distinct subtype of iMCD was first described in Japan, characterized by thrombocytopenia, anasarca, myelofibrosis, renal dysfunction, and organomegaly, known as TAFRO syndrome [3]. TAFRO has mostly been described in Japanese populations, with only a few cases being reported in Caucasians [4-5]. Fajgenbaum et al. proposed the first consensus diagnostic criteria for iMCD in 2017, which requires that certain conditions mimicking iMCD be excluded prior to making the diagnosis. These conditions include autoimmune diseases (such as systemic lupus erythematous and rheumatoid arthritis), infections (specifically HHV-8 and human immunodeficiency virus (HIV)), lymphomas, and POEMS syndrome [6]. The spectrum of iMCD presentation varies a great deal, from mild constitutional symptoms to multi-system organ failure secondary to hypercytokinemia.

The cytokine storm that drives the pathogenesis of TAFRO has yet to be completely understood. It has been hypothesized to be driven by an autoimmune, autoinflammatory, infectious, or paraneoplastic mechanism [7]. Several driver cytokines have been implicated thus far, including VEGF, IL-2, and IL- 6. IL-6 has specifically been linked to TAFRO, and was noted to be elevated in our patient’s case. The role of IL-6 is to induce B-cell and plasma cell maturation, acute inflammation, and VEGF secretion [8]. While TAFRO has a distinct clinical presentation, little is known about how these cytokines cause specific organ dysfunction. The hypercytokinemia associated with TAFRO is known to cause dysfunction of the renal, hematologic, and hepatic systems; with endocrine dysfunction not yet to be formally associated [3].

To our knowledge, this is a unique case of a patient diagnosed with TAFRO demonstrating multiple endocrinopathies. Our patient had significant disruption of multiple endocrine systems including thyroid, parathyroid, and androgen axes. Endocrinopathy has only been reported in a few cases in the literature. Oka et al. reported 2 patients with TAFRO demonstrating endocrinopathy, specifically hypothyroidism [9]. They reported decreasing VEGF levels with clinical remission following thyroid hormone replacement therapy, postulating that VEGF is a potential driver of endocrinopathy in these patients. Elevated VEGF levels have been surmised to be involved with precipitating endocrinopathy via angiogenic disruption in patients with POEMS [10]; however, our patient had normal VEGF levels. The multiple endocrinopathies that were present in our patient’s case appear to be a unique clinical finding; although, this specific organ dysfunction was not consistently investigated in previous cases.

The diagnosis of TAFRO syndrome requires a constellation of clinical and laboratory findings, along with an extensive workup for the exclusion of other diseases [6]. In order to establish a diagnosis of TAFRO syndrome, both major criteria and at least 2 minor criteria must be met. Our patient demonstrated both the major criteria of histopathological iMCD lymph node features and diffuse lymphadenopathy; along with the minor criteria of anemia, thrombocytopenia, hypoalbuminemia, elevated CRP, renal dysfunction, hepatosplenomegaly, ascites, and constitutional symptoms. These findings confirmed the diagnosis of TAFRO syndrome in our patient after a comprehensive workup excluded other autoimmune, infectious, and malignant conditions.

Due to the rarity of this disease and lack of clinical studies, treatment guidelines for this disease are still being investigated. Current evidence suggests that initial treatment with siltuximab and corticosteroids, with or without combination chemotherapy offer the best results. It is recommended to continue siltuximab indefinitely in patients who respond to initial treatment [1]. Given our patient’s complex and severe presentation, first-line therapy with anti-IL-6 siltuximab was initiated. A notable improvement was seen following 4 months of therapy, as evidenced by normalization of CRP, hemoglobin, albumin, renal function, and resolution of his endocrinopathy. Hypothyroidism resolved after treatment with levothyroxine; however, hypogonadism and hyperprolactinemia normalized after anti-IL-6 therapy. Due to our patient’s previously healthy state and resolution of his endocrinopathy with siltuximab treatment, we postulate that his endocrinopathy was a result of the excessive cytokine storm that drives TAFRO pathogenesis. Clinical remission has been maintained for 6 months on siltuximab monotherapy.

## Conclusions

The diagnosis of TAFRO remains challenging due to its rare occurrence and complex clinical presentation. Therefore, consistent and inclusive defining features of this novel entity are needed to assist in establishing diagnostic criteria and therapeutic strategies. We report a case of a Caucasian male with TAFRO syndrome and the unique findings of endocrinopathy to further characterize clinical presentations of this new entity.
